# Differentiating Childhood-Onset Schizophrenia From Other Childhood Disorders

**DOI:** 10.7759/cureus.22594

**Published:** 2022-02-25

**Authors:** Samicchya Adhikari, Nejla Ghane, Marisa Ascencio, Tiffany Abrego, Kapil Aedma

**Affiliations:** 1 Psychiatry, UnityPoint Health-Methodist Hospital/University of Illinois College of Medicine, Peoria, USA; 2 Psychiatry, University of Illinois College of Medicine, Peoria, USA

**Keywords:** childhood-onset schizophrenia, intellectual disability, major depressive disorder with psychotic features, autism spectrum disorder, schizophrenia

## Abstract

Childhood-onset schizophrenia (COS) is a rare disorder in which symptoms of schizophrenia occur before the age of 13 years. This disorder often has a complicated presentation that can mimic other childhood disorders including post-traumatic stress disorder (PTSD), autism spectrum disorder (ASD), major depressive disorder (MDD) with psychosis, and generalized anxiety disorder (GAD) among others. This is further complicated by the low prevalence rate of COS which limits understanding of the disorder. Accurate and timely diagnosis is crucial as failure to do so has adverse implications for long-term treatment outcomes and prognosis. In this study, a rare case of a 12-year-old girl with childhood-onset schizophrenia and key findings that help differentiate it from other childhood disorders are reviewed to guide diagnosis and treatment.

## Introduction

The prevalence of early-onset schizophrenia (symptoms occurring before the age of 18 years) is estimated to be about 0.5 % worldwide [1]. Very early-onset schizophrenia, also known as childhood-onset schizophrenia (COS), with symptom onset at age less than 13 years is even rarer, and the worldwide prevalence is unknown. However, the United States’ estimated prevalence is around 0.04% in the general population [1]. The rarity of the disorder presents obstacles in gathering data about the condition, and diagnosis is further complicated by other conditions that can closely mimic the symptoms of schizophrenia. 

Although the diagnostic criteria for schizophrenia in childhood are the same as those in adults, diagnosing children with schizophrenia can be challenging due to overlapping symptoms with other disorders and the differences in how children communicate their symptoms. Diagnoses that often become challenging to distinguish include post-traumatic stress disorder (PTSD), autism spectrum disorder (ASD), major depressive disorder (MDD) with psychosis, and generalized anxiety disorder (GAD). Medical conditions can also mimic symptoms, including seizure disorder, delirium, encephalitis, autoimmune disorders, metabolic disorders, among others [1]. 

In this study, a case of a 12-year-old girl with a current diagnosis of COS is discussed to help distinguish it from other childhood disorders that have overlapping symptoms. Differential diagnoses such as ASD, anxiety, PTSD, and MDD with psychosis are considered. COS diagnosis can be further complicated due to other comorbid psychiatric disorders.

## Case presentation

The patient is a 12-year-old biracial female with no known medical or psychiatric history. At baseline, the patient was described by her mother as cooperative albeit lower functioning but with no prior evidence of psychosis or behavioral issues. She had an IEP in school but was functioning and adapting well in school and home. She was first admitted to inpatient psychiatry at age of 11 years in July 2019 due to paranoia, delusions, social withdrawal, and bizarre behaviors. 

The patient’s father left abruptly in February 2019, and shortly thereafter, the patient expressed depressed moods, withdrew from friends and family, and was crying frequently. In July 2019, she began to stay awake at night and kept a knife under her pillow due to the feeling that someone was outside her home with a pickaxe. One night, she carried her four-year-old brother downstairs in the middle of the night due to her paranoid delusions progressing. She was subsequently hospitalized and diagnosed with major depressive disorder with psychotic features. She was started on risperidone (titrated to 1mg twice daily) and sertraline (titrated to 50mg daily) to target psychosis and depression. She responded well to this regimen and was followed in the outpatient setting.

The patient gained 80 pounds on risperidone, and it was therefore discontinued. Ziprasidone was started, which was titrated to 40mg twice daily. Unfortunately, ziprasidone was inefficacious, and she again became symptomatic with delusions (believed she lived in England and intermittently spoke in a British accent), hallucinations (observed responding to internal stimuli), and thought disorganization. She also began showing signs of obsessive-compulsive disorder. She shaved her eyebrows, was taking multiple daily showers, and engaged in excessive hand washing. Subsequently, she was re-hospitalized in December 2019. During this admission, she was diagnosed with obsessive-compulsive disorder due to her desire to avoid germs and her compulsive behaviors noted above, and unspecified psychosis. She was continued on sertraline and switched back to risperidone which was titrated again to 1mg twice daily. She responded relatively well and was subsequently discharged. 

She was re-hospitalized in April 2020 for 26 days due to worsening agitation, aggression (physically aggressive towards mother, hitting her), hallucinations, speech disorganization, and obsessions secondary to medication non-compliance. She was started on oral risperidone while titrating her Risperdal Consta long-acting injection to 50 mg bi-weekly. Unfortunately, the patient showed minimal improvement and was hospitalized again shortly after that in May 2020 for 37 days due to similar symptoms, including worsening aggression, hallucinations, and bizarre behaviors. Her lab work during prior hospitalizations, including complete blood count (CBC), complete metabolic panel (CMP), thyroid-stimulating hormone (TSH), urine analysis (U/A), urine drug screen (UDS) were within normal limits (Table 1). All prior physical examinations were also largely unremarkable. During this hospitalization, further medical workup was conducted since there was minimal response to the therapeutic dose of antipsychotics. Detailed medical workup was completed during the hospitalization in May 2020 (Table 1).

**Table 1:** Patient medical workup Ab: antibody; AGNA-1: anti-glial nuclear antibody type 1; CSF: cerebrospinal fluid; AMPA-R: alpha-amino-3-hydroxy-5-methyl-4-isoxazolepropionate receptor; CBA: cell-based assay; ANA: antinuclear antibody; ANNA: anti-neuronal nuclear antibody; CASPR2: anti-contactin-associated protein-like 2; CRMP-5-IgG: collapsin response mediator protein 5, IgG subtype; CRP: c-reactive protein; DPPX: dipeptidyl-peptidase-like protein type 6; IFA: indirect fluorescent antibody; dsDNA: double-stranded deoxyribonucleic acid; ESR: elevated sedimentation rate; GABA-B-R: gamma-aminobutyric acid B receptor; GAD65: glutamic acid decarboxylase 65; GFAP: glial fibrillary acidic protein; IgLON5: immunoglobulin-like cell adhesion molecule 5; LGI1 IgG: leucine-rich glioma inactivated protein 1, IgG subtype; MGLUR: metabotropic glutamate receptor; NIF: neuronal intermediate filament; NMDA-R: N-methyl-D-aspartate receptor; PCA: Purkinje cell cytoplasmic antibody; TR: anti-Tr antibody; RBC: red blood cell count; RPR: rapid plasma reagin; SS-B/La: Sjögren’s syndrome B and La antibodies; U/A: urine analysis; UDS: urine drug screen

Lab	Patient Value	Reference Range
AGNA-1, CSF	<1:2 titer (negative)	<1:2 titer
Aminolevulinic acid, plasma	<0.2 nmol/mL	<0.5 nmol/mL
AMPA-R Ab CBA, CSF	Negative	Negative
Amphiphysin Ab, CSF	<1:2 titer (negative)	<1:2 titer
ANA	<1:80 titer (positive)	<1:40 titer
ANNA-1, CSF	<1:2 titer (negative)	<1:2 titer
ANNA-2, CSF	<1:2 titer (negative)	<1:2 titer
ANNA-3, CSF	<1:2 titer (negative)	<1:2 titer
ASO	<13 (IU)/mL	0-408 (IU)/mL
CASPR2 IgG CBA, CSF	Negative	Negative
Ceruloplasmin	23 mg/dL	20-60 mg/dL
Copper, serum	0.94 mcg/mL	0.75-1.45 mcg/mL
Coproporphyrin, urine	46 nmol/L	<110 nmol/L
Cortisol, AM	14 mcg/dL	4 TO 19 mcg/dL
CRMP-5-IgG	<1:2 titer (negative)	<1:2 titer
CRP	<0.3 mg/dL	0-0.4 mg/dL
DPPX Ab IFA	Negative	Negative
dsDNA Ab	<1.0 (IU)/mL	<4.1 (IU)/mL
ESR	35 mm/h	0-19 mm/h
GABA-B-R Ab CBA, CSF	Negative	Negative
GAD65 Ab assay, CSF	0.00 nmol/L	≤0.02 nmol/L
GFAP IFA, CSF	Negative	Negative
Glucose, CSF	54 mg/dL	40-70 mg/dL
Heptacarboxylporphyrins, urine	<1 nmol/L	<7 nmol/L
Hexacarboxylporphyrins, urine	<1 nmol/L	<2 nmol/L
IgLON5 IFA, CSF	Negative	Negative
Lead	<0.3 mcg/dL	<5.0 mcg/dL
LGI1 IgG CBA, CSF	Negative	Negative
MGLUR1 Ab IFA, CSF	Negative	Negative
NIF IFA, CSF	Negative	Negative
NMDA-R CBA, CSF	Negative	Negative
PCA-1, CSF	<1:2 titer (negative)	<1:2 titer
PCA-2, CSF	<1:2 titer (negative)	<1:2 titer
PCA-TR, CSF	<1:2 titer (negative)	<1:2 titer
Porphobilinogen, plasma	<0.1 nmol/mL	<0.5 nmol/mL
Porphobilinogen, urine	0.3 mcmol/L	≤1.3 mcmol/L
Protein, CSF	27.3 mg/dL	12-60 mg/dL
RBC, CSF	0.00 units/mm	0.00 units/mm
Ribosomal P protein Ab	<0.2 Al	<1.0 AI
RPR	Negative	Negative
SS-B	2.8 AI	<1.0 AI
T4	0.8 ng/dL	0.7-1.9 ng/dL
TSH	1.432 mlU/L	0.300-5.000 mIU/L
Thyroglobulin Ab	116 IU/ML	10-60 IU/ML
Thyroperoxidase Ab	3149 IU/ML	15-60 IU/ML
Thyrotropin receptor Ab	<1.00 IU/L	0.00-1.75 IU/L
U/A		
UDS	Negative	Negative
Urinary copper, 24 hour	9 mcg/24 hours	Not established for this age range
Uroporphyrin, urine	3 nmol/L	≤30 nmol/L

All of the workup was unremarkable except positive ANA and elevated ESR. She also tested positive for Sjögren’s syndrome B and La antibodies (SS-B/La). Rheumatology was consulted and they concluded that positive SS-B/La were nonspecific due to a lack of evidence of multisystem disease. Video EEG and brain MRI were also conducted with no remarkable findings.

Endocrinology was also consulted as she was found to have elevated thyroid peroxidase (TPO) Ab and thyroglobulin antibodies, but the TSH and T4 were normal (Table 1). The endocrinologist diagnosed her with euthyroid Hashimoto’s thyroiditis but did not recommend pharmacological intervention. Outpatient follow-up with intermittent monitoring of thyroid levels (every six months) was recommended to determine the need for treatment.

Extensive psychological testing was also performed to evaluate intellectual functioning and psychosis. The patient had deficits across all domains in IQ testing and demonstrated significant deficits in working memory and processing speed indicating sluggish cognitive tempo, often associated with executive function deficits. Her full-scale IQ score was 70, and the diagnosis of moderate intellectual disability was given. During the evaluation of her psychosis, the patient was described as having odd eye contact, wavering between intensely staring and no eye contact at all. She was often non-responsive to questions and was observed responding to internal stimuli. The patient had also intermittently talking in a British accent and became incoherent at times. Responses were often unrelated to the questions being asked. 

**Figure 1:**
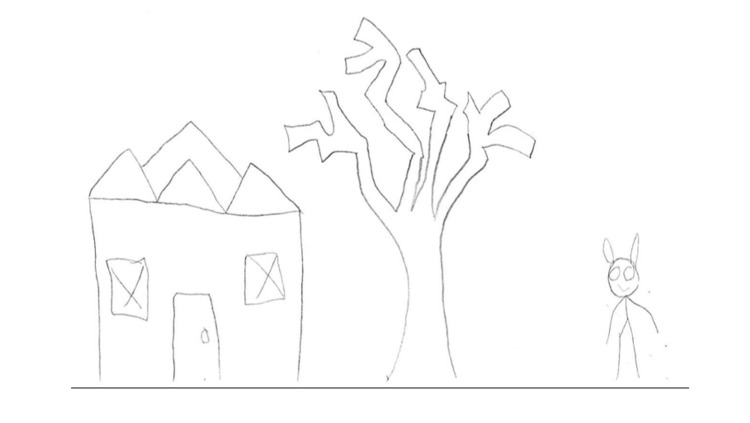
This drawing was completed by the patient after a prompt to draw a house, a tree, and a person

During a rapport-building exercise, she was asked to draw a house, a tree, and a person. Her drawings were described as haphazard and had many details as an afterthought. Figure 1 shows her drawings and a more thorough explanation of her drawing process. She was given personality self-reports that indicated she was suffering from severe depression, paranoia, and hallucinations. After reviewing her entire medical record, psychological testing, and behavioral observations, the psychiatrist and psychologist gave her the provisional diagnosis of schizophrenia pending rule-out of medical causes (some of the medical workup results above were still pending at the time of psychological evaluation). 

Patient’s background, developmental, social, and family history

The patient was born full-term, but the pregnancy was complicated by uncontrolled gestational diabetes. The patient’s mother was unaware of the pregnancy until 20 weeks of gestation, which delayed prenatal care. The mother also took lorazepam daily during pregnancy. The patient was born via cesarean section necessitated by macrosomia. At birth, the patient was lethargic and did not cry.

The patient had significant delays in developmental milestones, with crawling at 16 months, walking at 24 months, speaking at four years, and toilet training at four years. She received speech, occupational, and physical therapy starting in pre-school. She was evaluated for autism in early childhood, which was negative, but she was diagnosed with developmental delay. She had an individualized education program (IEP) throughout the school. 

In terms of family history, the patient's mother suffers from anxiety and depression. She has a paternal aunt with schizoaffective disorder. As for her social history, she lives with her mother and her two older brothers (14 and 16 years old). As noted above, her father (who she was reportedly very close to) left abruptly from her life in February of 2019, and symptoms subsequently began. During grade school and middle school, the patient experienced bullying but otherwise has not experienced any major trauma, including physical, sexual, or emotional abuse. She lives in a safe neighborhood with no known exposure to violence.

## Discussion

As discussed above, the patient had an extensive workup that included psychiatric, psychological, and medical evaluations until finally coming to a COS diagnosis. Early-onset schizophrenia is characterized by positive and negative psychotic symptoms that begin between the age of seven and 13 years, and influence normal development and functioning. Auditory, visual, and tactile hallucinations are more commonly seen in COS than delusions. The patient in this case had both delusions and hallucinations. Other notable COS symptoms include language, motor, and social delays, which were observed in this patient [2]. Interestingly, several studies have found evidence of infantile autism or other pervasive developmental disorders (PDD) three to five years before the onset of psychotic symptoms in patients with COS [1,3]. Neuromotor, receptive language and cognitive developmental impairments are notable ASD/PDD symptoms experienced before a COS diagnosis [1,4]. Furthermore, the neurocognitive deficiencies seen in patients with ASD may be a risk factor for future psychosis based on one case report [5]. There have been reports that the actual prevalence of COS-ASD comorbidity is less than 4% [4].

In terms of brain imaging, it has been well established that children with ASD have an increased head size and brain volume within the first three years of life. This indicates an excess of early postnatal brain development. Children with COS exhibit a loss of gray matter through adolescence, which implies excess in the brain maturation process. Interestingly, both the rate of increase in brain volume seen in ASD and loss of gray matter seen in COS decrease with age, the acceleration ultimately normalizing in adulthood [6]. There is also family and genetic evidence connecting the two disorders, including birth complications as a predisposing factor [4,6]. The patient’s mother did not receive prenatal care until 20 weeks of gestation, took lorazepam, and had gestational diabetes, which may have contributed to this patient’s symptom development. There have been studies linking maternal diabetes, especially gestational diabetes with ASD in the offspring [7]. There have also been studies that have linked diabetes in pregnancy with risk of schizophrenia in the offspring [8]. Benzodiazepine use in pregnancy can affect neurodevelopment and has been associated with various facial anomalies (not seen in our patient), intellectual disability, and developmental delays [9].

The differences between ASD and COS are frequently subtle due to the young-onset and overlap of symptoms. Some patients with ASD exhibit psychotic reactions to triggering situations and can be misconstrued as a separate psychotic diagnosis like COS [5]. Similarly, the withdrawn nature of patients with COS may be ascribed to ASD. Psychotic features such as hallucinations seen in COS can be confused with nightmares or tantrums, thus causing a completely missed COS diagnosis. This is an important consideration because a child with an early ASD diagnosis may not receive a COS diagnosis at a later age, even if new positive or negative symptoms appear. In either case, the patient may be under-, over-, or mistreated. A delay in treatment, pharmacological or otherwise, can result in an increased likelihood of hospitalization [4]. The patient, in this case, had an evaluation for ASD at an early age that ruled out the diagnosis. These may have been early signs of COS that were unidentified until they became more severe. 

A brief discussion regarding intellectual disability and psychosis is also pertinent in this case. Although the symptoms of psychosis are the same in patients with intellectual disability, including positive symptoms like delusions, hallucinations, and disorganized/bizarre speech or behavior, the diagnosis can be more difficult in that symptoms are based on internal experiences which is difficult to reliably diagnose in people who are non-verbal or profoundly intellectually disabled due to their limited communication skills. However, our patient showed a clear and notable change from her typical baseline functioning as reported by the family. Prior to her first admission, she did not express any paranoia and was never observed responding to internal stimuli which supported a COS diagnosis.

There is an increasing curiosity regarding the complex relationship between trauma and psychosis. Several studies have shown the prevalence of post-traumatic stress disorder (PTSD) to be three times more likely in individuals with a severe mental illness when compared to the general population. Researchers who studied this direct correlation found that exposure to trauma affects similar neural pathways, which predispose individuals to common mental health disorders, such as PTSD and schizophrenia [10]. Before the addition of PTSD into the Diagnostic and Statistical Manual of Mental Disorders III (DSM-III), it was not uncommon for veterans to be misdiagnosed with schizophrenia due to various similarities in clinical presentation, including aggression, irritability, nightmares, and decreased level of functioning [10].

Regarding clinical presentation, flashbacks of traumatic events in those with PTSD may be hard to differentiate from the hallucinations seen in COS. Additionally, severe PTSD can manifest itself as paranoia and hallucinations, both hallmark symptoms present in COS. A systematic review that studied clinical characteristics of hallucinations found that PTSD and schizophrenia both caused auditory hallucinations of negative voices originating in external space [11]. Similarly, a subtype of PTSD characterized by dissociative and depersonalization symptoms, resembles common manifestations of schizophrenia, such as auditory hallucinations, thought broadcast, thought withdrawal, and delusional perception [12]. Finally, the deterioration of social or occupational functioning seen in PTSD can be similar to the decreased level of interpersonal, academic, or social functioning found in COS as well as various other psychiatric disorders.

The diagnoses of anxiety disorder (AD) and COS are similar in the sense that children with both perceive a threat by the world around them and are hypervigilant toward those threatening stimuli [13,14]. Symptoms of AD that may be similar to COS include irritability, obsession/compulsion, and difficulty concentrating such as that seen in our patient. GAD symptoms can include excessive worry about multiple activities that lead to one or more associated symptoms. In comparison to COS, the worries that children with GAD surround present or future occurrences in their lives such as school performance. Our patient, however, had worried about unlikely incidents, such as a man outside with a pickaxe. Children with social anxiety disorder (SAD) exhibit worry about surrounding social interactions and the possible reactions that the child may have to that worry. These children may react to this anxiety by crying, freezing, or failing to speak. In some cases, the feelings of anxiety may lead to agoraphobia or fear of being in situations that they cannot escape. Although fear of being observed by others is a key part of SAD and COS, patients with COS have unrealistic delusions about who may be observing them and why.

It is important to remember that auditory and visual hallucinations, delusions, and other symptoms of psychosis are not a normal presentation of a child with an AD, whereas they are diagnostic criteria for COS. Children with COS may well exhibit signs and symptoms of anxiety that lead to distress in the individual. A systematic review found that an average of 50% of patients with schizophrenia exhibit significant anxious symptoms [15]. It is important to note that a sample of patients with schizophrenia, albeit adults, showed a relatively high rate of co-current AD, including SAD (17%), obsessive-compulsive disorder (13%) which was included among the anxiety disorders in previous editions of the DSM, and GAD (12%) [16]. With proper rapport, history taking, and assessments, a practitioner should be able to differentiate the root cause of the anxious symptoms associated with COS and categorize the patient as co-morbid or not.

A study conducted by the National Institute of Mental Health (NIMH) found that 33 cases who were initially diagnosed with COS yielded a diagnosis of a psychotic mood disorder after an extensive screening of 215 total pediatric patients [17]. Although psychosis is present in both COS and MDD with psychotic features, the distinguishing factor is the temporal relationship of the psychotic episodes. The psychotic symptoms in MDD with psychotic features are episodic and occur only during an episode of major depression, while the psychotic symptoms in patients with schizophrenia occur in the absence of an identifiable mood disorder [18].

Differentiating the flat affect seen in schizophrenia from the depressive symptoms of MDD can be challenging [19]. Determining when the negative symptoms or withdrawal symptoms subside is helpful. Children with COS may not experience resolution of their negative symptoms, while children with MDD with psychotic features will most likely experience improvement of mood symptoms. Additionally, the decreased energy seen in COS compared to the psychomotor retardation of depression present another challenge in the complex diagnostic process [20]. Finally, it is important to consider that comorbidity is high in schizophrenia, with an estimated 50% rate of comorbid depression [21].

Providers can also use several biomarkers to distinguish schizophrenia from MDD with psychotic features. Examples of such biomarkers include the hypothalamic-pituitary-adrenal axis (HPA) activity [22]. One study found that patients with psychotic depression had higher basal evening cortisol compared to patients with schizophrenia (who had similar levels with controls) [22]. While the patient’s TSH was unremarkable, no other biomarkers of the HPA axis were investigated.

This case presentation highlights several factors that led to the development of this patient's schizophrenia as well as her intellectual disability. The specific factors include her mother's lack of prenatal care, prenatal exposure to lorazepam, history of uncontrolled gestational diabetes, and birth complications which as described above can play a role in the development of schizophrenia as well as intellectual disability. She also carried a family history of an aunt with schizoaffective disorder. In addition, she had several social stressors that contributed to her first psychotic break. She was bullied in school, and when her father left, her psychotic symptoms followed shortly after this abrupt and distressing change. This case was diagnostically complicated due to overlapping symptoms of various disorders and also due to the patient's co-morbid intellectual disability. However, this case is most consistent with childhood-onset schizophrenia due to both the positive and negative symptoms that were observed that cannot quite be explained by the other diagnoses. These included observations of responding to internal stimuli, vivid descriptions of paranoid delusions and hallucinations despite her intellectual disability, and observed negative symptoms of social withdrawal, anhedonia, flat affect, and clear decompensation from her baseline. The combination of these factors and symptoms makes COS the most likely diagnosis.

## Conclusions

Diagnosing COS presents many challenges. Several other childhood disorders such as autism spectrum disorder, post-traumatic stress disorder, anxiety disorders, and major depressive disorder with psychotic features have symptoms that overlap with COS, and misdiagnosis is common. Obtaining a thorough history, not only with regards to overt symptoms but also in terms of the temporal relationship of symptoms, developmental, social, and family history is essential. A detailed understanding of the differential diagnoses and the underlying similarities and key distinguishing features is imperative. A comprehensive medical evaluation is also required as organic medical illnesses such as seizure disorder, delirium, encephalitis, an autoimmune disorder, metabolic disorders, among others, can also present with psychotic symptoms. Identifying children with schizophrenia is further complicated as many children also have other underlying comorbid psychiatric conditions. Furthermore, the rare nature of COS and unfamiliarity can deter providers from making this diagnosis, even when suspected. However, timely diagnosis and treatment are crucial as failure to do so has adverse implications for long-term treatment outcomes and prognosis.

## References

[1] Alaghband-Rad J, Mckenna K, Gordon CT (1995). Childhood-onset schizophrenia: the severity of premorbid course. J Am Acad Child Adolesc Psychiatry.

[2] Remschmidt H, Theisen FM (2005). Schizophrenia and related disorders in children and adolescents. Neurodevelopmental Disorders.

[3] Watkins JM, Asarnow RF, Tanguay PE (1988). Symptom development in childhood onset schizophrenia. J Child Psychol Psychiatry.

[4] Galli Carminati G, Tagan C, Zecca G, Carminati F (2018). Between autistic spectrum disorder (ASD) and childhood onset schizophrenia (COS): a proposal for a passerella syndrome. Neuropsychiatry.

[5] Galli Carminati G, Crettol C, Carminati F (2017). Between autistic spectrum disorder (ASD) and childhood onset schizophrenia (COS): a case report. Neuropsychiatry.

[6] Rapoport J, Chavez A, Greenstein D, Addington A, Gogtay N (2009). Autism spectrum disorders and childhood-onset schizophrenia: clinical and biological contributions to a relation revisited. J Am Acad Child Adolesc Psychiatry.

[7] Wan H, Zhang C, Li H, Luan S, Liu C (2018). Association of maternal diabetes with autism spectrum disorders in offspring: a systemic review and meta-analysis. Medicine (Baltimore).

[8] Cannon M, Jones PB, Murray RM (2002). Obstetric complications and schizophrenia: historical and meta-analytic review. Am J Psychiatry.

[9] Bercovici E (2005). Prenatal and perinatal effects of psychotropic drugs on neurocognitive development in the fetus. J Dev Disabil.

[10] Seedat S, Stein MB, Oosthuizen PP, Emsley RA, Stein DJ (2003). Linking posttraumatic stress disorder and psychosis: a look at epidemiology, phenomenology, and treatment. J Nerv Ment Dis.

[11] Waters F, Fernyhough C (2017). Hallucinations: a systematic review of points of similarity and difference across diagnostic classes. Schizophr Bull.

[12] Huppert JD, Smith TE (2005). Anxiety and schizophrenia: the interaction of subtypes of anxiety and psychotic symptoms. CNS Spectr.

[13] Bar-Haim Y, Lamy D, Pergamin L, Bakermans-Kranenburg MJ, van IJzendoorn MH (2007). Threat-related attentional bias in anxious and nonanxious individuals: a meta-analytic study. Psychol Bull.

[14] Langarita-Llorente R, Gracia-Garcia P (2019). Neuropsychology of generalized anxiety disorders: a systematic review. [Article in Spanish]. Rev Neurol.

[15] Pokos V, Castle DJ (2006). Prevalence of comorbid anxiety disorders in schizophrenia spectrum disorders: a literature review. Curr Psychiatry.

[16] Cosoff SJ, Hafner RJ (1998). The prevalence of comorbid anxiety in schizophrenia, schizoaffective disorder and bipolar disorder. Aust N J Psychiatry.

[17] Calderoni D, Wudarsky M, Bhangoo R (2001). Differentiating childhood-onset schizophrenia from psychotic mood disorders. J Am Acad Child Adolesc Psychiatry.

[18] Rothschild AJ (2013). Challenges in the treatment of major depressive disorder with psychotic features. Schizophr Bull.

[19] Carlson GA, Bromet EJ, Sievers S (2000). Phenomenology and outcome of subjects with early- and adult-onset psychotic mania. Am J Psychiatry.

[20] Courvoisie H, Labellarte MJ, RiddleMA RiddleMA (2001). Psychosis in children: diagnosis and treatment. Dialogues Clin Neurosci.

[21] Buckley PF, Miller BJ, Lehrer DS, Castle DJ (2009). Psychiatric comorbidities and schizophrenia. Schizophr Bull.

[22] Cherian K, Schatzberg AF, Keller J (2019). HPA axis in psychotic major depression and schizophrenia spectrum disorders: Cortisol, clinical symptomatology, and cognition. Schizophr Res.

